# Determinants of serum levels of vitamin D: a study of life-style, menopausal status, dietary intake, serum calcium, and PTH

**DOI:** 10.1186/1472-6874-13-33

**Published:** 2013-08-15

**Authors:** Leila Shirazi, Martin Almquist, Johan Malm, Elisabet Wirfält, Jonas Manjer

**Affiliations:** 1 Department of Surgery, Ystad Hospital, Ystad, Sweden; 2 Department of Clinical Sciences Malmö Surgery, Lund University, Malmö, Sweden; 3 Department of Surgery, Skåne University Hospital, Malmö/Lund, Sweden; 4 Department of Laboratory Medicine, Section for clinical chemistry, Lund University, Malmö, Sweden; 5 Department of Clinical Sciences Malmö Nutritional Epidemiology, Lund University, Malmö, Sweden; 6 Department of Plastic Surgery, Skåne University Hospital, Malmö, Sweden

## Abstract

**Background:**

Low blood levels of vitamin D (25-hydroxy D3, 25OHD3) in women have been associated with an increased risk of several diseases. A large part of the population may have suboptimal 25OHD3 levels but high-risk groups are not well known. The aim of the present study was to identify determinants for serum levels of 25OHD3 in women, i.e. factors such as lifestyle, menopausal status, diet and selected biochemical variables.

**Methods:**

The study was based on women from the Malmö Diet and Cancer Study (MDCS), a prospective, population-based cohort study in Malmö, Sweden. In a previous case–control study on breast cancer, 25OHD3 concentrations had been measured in 727 women. In these, quartiles of serum 25OHD3 were compared with regard to age at baseline, BMI (Body Max Index), menopausal status, use of oral contraceptives or menopausal hormone therapy (MHT) , life-style (e.g. smoking and alcohol consumption), socio-demographic factors, season, biochemical variables (i.e. calcium, PTH, albumin, creatinine, and phosphate), and dietary intake of vitamin D and calcium. In order to test differences in mean vitamin D concentrations between different categories of the studied factors, an ANOVA test was used followed by a t-test. The relation between different factors and 25OHD3 was further investigated using multiple linear regression analysis and a logistic regression analysis.

**Results:**

We found a positive association between serum levels of 25OHD3 and age, oral contraceptive use, moderate alcohol consumption, blood collection during summer/ autumn, creatinine, phosphate, calcium, and a high intake of vitamin D. Low vitamin D levels were associated with obesity, being born outside Sweden and high PTH levels.

**Conclusions:**

The present population-based study found a positive association between serum levels of 25OHD3 and to several socio-demographic, life-style and biochemical factors. The study may have implications e. g. for dietary recommendations. However, the analysis is a cross-sectional and it is difficult to suggest Lifestyle changes as cause- effect relationships are difficult to assess.

## Background

Low blood levels of vitamin D (25-hydroxy D3, 25OHD3) in women have been associated with an increased risk of hypertension [1], myocardial infarction [2], sudden cardiac death [3], depression [4], diabetes [5], obesity [6], fracture [7] and cancer [8,9]. However, determinants of levels of 25OHD3 are not well known, and few studies have examined the impact of lifestyle, and menopausal status, dietary and biochemical factors on vitamin D status. Furthermore, a large part of the population in northern countries might suffer from suboptimal 25OHD3 levels because of inadequate exposure to sunlight [10], due to geographical location [11], cultural differences [12], and race [13,14].

Sun exposure plays a central role in vitamin D metabolism as vitamin D is formed in the skin under the influence of UV light [15], and there is a well-known seasonal variation with lowest levels during the winter [16]. The other source of vitamin D is from the diet, but the association between dietary intake and serum 25OHD3 levels has not been extensively investigated [17]. Sun exposure and diet may indeed be affected by socioeconomic factors and life-style; for instance low vitamin D levels have been noted among low income groups [18], and among individuals with heavy alcohol consumption [19]. However, apart from this, few studies have reported on vitamin D in relation to life-style and menopausal status. Vitamin D metabolism is closely related to PTH, calcium and phosphate [20].

This study is based on data from the Malmö Diet and Cancer Study (MDCS), a prospective, population-based cohort study in Malmö, Sweden [21]. At baseline, 17,035 women answered a questionnaire on life-style, reproductive life and socio-demographic factors. An important part of the examination was a dietary assessment and collection of blood samples [22].

The aim of the present study was to identify determinants for serum levels of 25OHD3 in women, i.e. factors such as lifestyle, menopausal status, diet and selected biochemical variables.

## Methods

### The malmö diet and cancer study

The Malmö Diet and Cancer Study (MDCS) is a prospective, population-based cohort study in Malmö, Sweden’s third largest city with about 300,000 inhabitants. The main aim of the MDCS was to estimate the potential association between dietary factors and malignancy [23]. All women in Malmö born 1923–1950 were invited and the baseline examination took place between 1991 and 1996. The majority of women were born in Sweden or other Nordic countries. Finally, 17,035 women participated, corresponding to a participation rate of approximately 40%.

### Baseline examination

All participants completed a questionnaire on socio-economic factors (e.g. education, occupation, country of birth), life-style (e.g. smoking, alcohol consumption, use of oral contraceptives (OC) and menstrual status), and for women also menopausal status. Smoking status was classified as current, never or ex-smoking. An open ended question assessed use of medications. For the purpose of the present analysis, menopausal hormone therapy (MHT) was classified into two categories: non-users and current users. Women were classified as pre- or peri/ postmenopausal using information on previous gynaecologic surgery and last menstruation as previously described in detail [24]. Anthropometric measurements were performed and Body Mass Index (BMI) was calculated (kg/m^2^). BMI was classified as underweight <20, normal weight ≥ 20 - <25, overweight ≥25 - <30, and obesity ≥30. Season was classified as “spring” (March-April), “summer” (May-August), “autumn” (September-October) and “winter” (November-February), based on the date of the baseline examination.

### Dietary data

Dietary data was obtained through a modified diet history consisting of (a) a 168-item dietary questionnaire for assessment of the consumption frequencies and portion sizes of regularly eaten foods (i.e. sandwiches, cakes and cookies, fruit, breakfast cereals, milk and yoghurt, coffee, tea, candy, snacks etc.) and the general meal pattern, (b) a 7 day menu-book for registration of cooked lunch and dinner meals, cold beverages including alcohol, drugs, natural remedies and nutrient supplements, and c) a one-hour diet history interview [25]. The food intake information was converted to nutrient intake data using the nutrient information available in the MDCS Food and Nutrient Database. This database, specifically developed for the MDCS, originates from PC KO ST2 -93 of the Swedish National Food Administration. The average daily intake of total energy and nutrients was calculated. The procedure has been previously described in detail [26]. The relative validity of the diet history method has previously been examined with 18 days of weighed food records collected during one year as the reference [25]. In women, the energy-adjusted validation correlation coefficients were for calcium 0.65 and for total energy intake 0.54, when administrating the diet history prior to the reference method.

This study examined the following nutrient variables: total energy (kcal), vitamin D (μg) and calcium (mg). Separate analyses were performed for intake related to food, and total intake including supplements. In order to adjust for energy, the residual method was applied using log-transformed variables of total energy, vitamin D and calcium intakes [26]. Four different variables were created by regressing the log-transformed variables separated on total energy intake: (1) vitamin D-dietary; (2) vitamin D-total (i.e. dietary and supplement); (3) calcium-dietary; and (4) calcium-total. The residuals were saved for each variable, and individuals were categorized into five categories (quintiles). The limits for the original variables were given for each quintile.

Alcohol consumption was divided into five categories: zero, low (<15 g alcohol/day), medium (15-30 g alcohol/day), high (>30 g alcohol/day), and infrequent use. The amount per day was obtained from the menu book. Participants with zero consumption in the menu book and with no consumption during the previous thirty days or previous year according to the questionnaire were categorized as zero consumers [27]. Participants with zero consumption in the menu book but reporting alcohol consumption during the previous thirty days or previous year were regarded as infrequent users. For example a glass of wine (12 centilitres) corresponds to 15 grams pure alcohol.

### Study population

The present study is based on data from female controls from a previous nested case–control study within the MDCS [22]. In that study, cases were women with incident breast cancer; controls were selected from MDCS subjects at risk at the time of disease occurrence of the case. Cases and controls were matched for age, calendar time of blood sampling, and menopausal [28] status. Out of 738 controls, 11 had no data on 25OHD3 concentrations and were excluded. Thus, the present study is based on data from 727 women.

### Blood samples and laboratory methods

Blood samples were drawn at baseline in non-fasting subjects by a trained nurse and plasma and serum separated within 1 hour. The samples were subsequently stored in a biological bank at −80°C before analysis [23]. Serum was retrieved from un-thawed samples in the MDCS bio-bank was analysed for 25OHD3, PTH, calcium, phosphate, creatinine and albumin. 25OHD3 was analysed with high pressure liquid chromatography (HPLC) and PTH with the Immulite® 2000 Intact PTH immunoassay (Diagnostic Products Corporation, Los Angeles, CA). Total calcium was analysed by neutral carrier ion-selective electrode [29], albumin by rate immunonephelometry [28], phosphate by a colorimetric method by complexing with ammoniummolybdate and creatinine by the Jaffé method. These analyses were carried out with the Synchron LX System (Beckman Coulter Inc., Brea, CA.). Inter-assay CVs were for 25OHD3 8.5% at 70 nmol/L and 7.1% at 210 nmol/L, for PTH 4% at 5.9 pmol/L and at 40.3 pmol/L, for calcium 2% at 2.00 mmol/L and at 3.10 mmol/L, for albumin 4% at 25 g/L and 2% at 48 g/L, for creatinine 12% at 34 μmol/L and 4% at 129 μmol/L, and for phosphate 3% at 0.66 mmol/L and 5% at 2.5 mmol/L. The Clinical Chemistry laboratory at Skåne University Hospital Malmö is accredited by Swedac (The Swedish Board for Accreditation and Conformity Assessment) and takes part in the external quality assurance program of Instand e.V., Düsseldorf, Germany.

### Statistical methods

Serum levels of 25OHD3 were normally distributed. The cohort subjects were ranked into quartiles based on serum levels of 25OHD3. Different quartiles of serum 25OHD3 were compared with regard to age at baseline (continuous and as categories in five-year intervals), life-style, BMI (continuous and categorical), menopausal status, socio-demographic factors, season, biochemical variables, and dietary intakes.

Means of serum 25OHD3 were calculated in different categories of all factors mentioned above. Confidence intervals were chosen as an estimation of the accuracy of the estimated means. In order to examine differences in mean vitamin D concentrations between different categories of the studied factors, ANOVA was used followed by a Bonferroni t-test, thus multiplying the obtained p-values with the number of comparisons. All tests were two-sided and a p-value of 0.05 was considered statistically significant. Missing values constituted less than 1% for all factors.

Serum 25OHD3 levels were dichotomized into low (≤75 nmol/L) and high (>75 nmol/L), i.e., the cut-off indicating the minimum level previously estimated to be sufficient [30]. Odds ratios with 95% confidence intervals (CI) were estimated for high versus low concentrations, in relation to each of the studied factors, using a logistic regression analysis. In a second model, the odds ratios for high versus low concentrations were adjusted for age, season and storage time. Age has previously been related to vitamin D levels and there is a well-known seasonal variation in vitamin D levels [31]. The potential effect of biological degradation is not well known and this justified the inclusion of storage time in the final model. In a sensitivity analysis, a third model included adjustment for total dietary vitamin D intake (entered as quintiles).

The relation between different factors and 25OHD3 was further investigated using multiple linear regression analysis. All categorical variables in the linear regression analysis were recorded and entered as multiple categorical variables with values as 0 or 1. Partial regression coefficients (βi) with 95% confidence intervals, adjusted for age, season and storage time, were reported. All statistical analyses were performed using SPSS version 17.0.

## Results

In this cross-sectional study on 727 women, mean age at blood sampling was 56.9 years (range: 41–73 years), 74% were postmenopausal, 91% were born in Sweden, mean BMI was 25.5 kg/m2 (SD: 16.61 – 50.19), and the mean level of 25OHD3 was 88.3 nmol/L (SD: 18–173).

Co-variates used in the multivariate analyses, (i.e., age at baseline, season and storage time), were all examined in relation to 25OHD3 concentrations and presented below. There was no significant association between storage time and 25OHD3 concentrations. The odds of high 25OHD3 concentration, adjusted for age and season, were for storage time as a continuous variable: 0.98 (0.87-1.09). The linear regression analysis showed an adjusted regression coefficient for storage time of −0.59 (−1.97 to 0.79). That is, there was no strong association between storage time and 25OHD3 levels.

### Age, life-style, menopausal status, season, and socio-demographic factors

Vitamin D quartiles 3 and 4 were characterised by a relatively large proportion of women >65 years old Table 1. The positive association between age and high vitamin D levels was confirmed with a trend of increase in vitamin D levels observed in older women in the analyses of mean 25OHD3 in different age categories, Table 2, and in the continuous linear regression analysis, Table 3. There was also a positive association between vitamin D levels and use of OC in all analyses, Tables 1, 2, and 3. Women with relatively high 25OHD3 levels were less often overweight or obese, and this negative association was also seen comparing mean levels in different BMI categories, Table 2, in the dichotomised logistic regression analysis, and in the linear regression analysis, Table 3.

**Table 1:** Distribution of serum vitamin D quartiles in relation to reproductive history, life-style factors and season

**Factor**	**Serum vitamin D quartile [nmol/L]**				
	**1 [18–69]**	**2 [70–86]**	**3 [87–104]**	**4 [105–173]**	**All**
	**n = 179**	**n = 187**	**n = 177**	**n = 184**	**n = 727**
Column percent*; *mean (SD) in italics*					
Age (years)	*56.2 (7.3)*	*56.2 (7.9)*	*58.1 (7.2)*	*57.2 (7.2)*	*56.9 (7.2)*
≤50	27.9	23.5	18.1	19.6	22.3
>50 – ≤55	22.3	23.0	18.6	26.1	22.6
>55 – ≤60	19.0	20.3	21.5	16.8	19.4
>60 – ≤65	17.9	21.9	20.9	19.6	20.1
>65	12.8	11.2	20.9	17.9	15.7
BMI (kg/m2)	*26.7 (4.96)*	*25.5 (4.5)*	*25.1 (3.4)*	*24.6 (3.4)*	*25.5 (4.2)*
<20	5.0	6.4	4.5	6.5	5.6
≥20 – <25	39.1	44.9	48.6	52.2	46.2
≥25 – <30	34.6	34.2	40.1	33.7	35.6
≥30	21.2	14.4	6.8	7.6	12.5
Oral contraception					
No	52.5	52.9	51.4	44.6	50.3
Yes	47.5	47.1	48.6	55.4	49.7
Menopausal status					
Pre	30.7	26.7	21.5	23.9	25.7
Peri/post	69.3	73.3	78.5	76.1	74.3
MHT** (n = 537)					
No	77.4	86.1	72.7	76.4	78.1
Yes	21.0	13.9	26.6	23.6	21.3
Smoking status					
Never	40.8	43.3	42.9	41.3	42.1
Current	29.6	24.1	29.4	27.7	27.6
Ex	29.6	32.6	27.7	31.0	30.3
Alcohol consumption					
Zero	11.7	6.4	7.9	5.4	7.8
<15 g/day	58.7	68.4	61.6	62.5	62.9
15 – 30 g/day	12.8	11.2	16.9	14.1	13.8
>30 g/day	3.9	1.1	2.8	3.3	2.8
Infrequent	12.8	12.8	10.7	14.7	12.8
Baseline season					
Spring	43.6	25.1	27.1	14.1	27.4
Summer	14.5	22.5	31.1	43..5	27.9
Autumn	15.6	22.5	20.3	23.4	20.5
Winter	26.3	29.9	21.5	19.0	24.2
Education					
O-level college	67.0	66.3	67.8	71.7	68.2
A-level college	10.1	7.0	7.9	6.5	7.8
University	22.9	26.7	24.3	21.7	23.9
Socio-economic index					
Manual	39.1	35.8	35.6	40.2	37.7
Non-manual	53.1	51.3	59.9	53.8	54.5
Employer	7.3	12.8	4.5	6.0	7.7
Born in Sweden					
Yes	83.2	91.4	93.8	92.9	90.4
No	16.8	8.6	6.2	7.1	9.6

* Due to missing values, percentages do not always add up to 100%. ** Only in peri/postmenopausal.

**Table 2:** Mean serum vitamin D levels in relation to reproductive history, life–style factors and season

**Factor**	**No.**	**Mean (95% CI)**	T–test (p–value*)	**ANOVA (p–value)**
Age (years)				
≤50	162	84.4 (80.3–88.6)	Ref.	
>50 – ≤55	164	88.6 (84.3–92.8)	0.10	
>55 – ≤60	141	87.7 (83.0–92.5)	0.29	
>60 – ≤65	146	88.5 (84.6–92.5)	0.13	
>65	114	93.6 (88.3–98.9)	0.01	0.107
BMI (kg/m2)				
<20	41	93.2 (83.6–102.8)	0.62	
≥20 – <25	336	91.0 (88.1–93.9)	Ref.	
≥25 – <30	259	87.9 (84.6–91.1)	0.16	
≥30	91	77.3 (71.7–82.9)	<0.001	<0.001
Oral contraception				
No	366	86.9 (84.2–89.5)	Ref.	
Yes	361	89.7 (86.8–92.6)	0.011	0.011
Menopausal status				
Pre	187	85.0 (81.1–88.8)	Ref.	
Peri/Post	540	89.4 (87.1–91.7)	0.056	0.056
MHT** (n = 537)				
No	422	89.2 (86.6–91.8)	Ref.	
Yes	115	90.8 (85.7–95.9)	0.57	0.27
Smoking status				
Never	306	87.9 (85.0–90.8)	Ref.	
Current	201	87.3 (83.3–91.3)	0.813	
Ex	220	89.7 (86.0–93.4)	0.443	0.635
Alcohol consumption				
Zero	57	80.3 (72.8–87.8)	Ref.	
<15 g/day	457	88.9 (86.4–91.4)	0.025	
15 – 30 g/day	100	89.5 (84.8–94.3)	0.032	
>30 g/day	20	82.7 (66.3–99.1)	0.762	
Infrequent	93	89.7 (83.9–95.5)	0.049	0.169
Baseline season				
Spring	199	78.6 (74.8–82.4)	Ref.	
Summer	203	99.0 (95.2–102.7)	<0.001	
Autumn	149	89.6 (85.8–93.5)	<0.001	
Winter	176	85.7 (81.8–89.6)	0.011	<0.001
Education				
O–level college	496	89.3 (86.9–91.7)	Ref.	
A–level college	57	83.4 (75.8–91.0)	0.128	
University	174	87.0 (83.0–91.0)	0.324	0.236
Socio–economic index				
Manual	274	89.0 (85.6–92.5)	Ref.	
Non–manual	396	88.5 (85.9–91.1)	0.796	
Employer	56	83.9 (77.3–90.5)	0.221	0.158
Born in Sweden				
Yes	657	89.3 (87.2–91.4)	Ref.	
No	70	78.6 (72.0 –85.3)	0.002	0.00

* Compared to reference (p–value). ** Only in peri/postmenopausal.

**Table 3:** **Crude and adjusted odds ratios (OR) for high (> 75 nmol/L) *vs*. low (≤75 nmol/L) vitamin D levels, and regression coefficients (βi) from multiple regression analysis, in relation to reproductive history, life–styles factors and season**

**Factor**	**Low (n)**	**High (n)**	**Crude OR (95% CI)**	Adjusted*OR (95% CI)	**βi (95% CI)**	Adjusted* βi (95% CI)
Age (years)						
≤50	59	103	1.00	1.00	Baseline	Baseline
>50 – ≤55	53	111	1.20 (0.76–1.90)	1.18 (0.73–1.92)	4.13 (–1.78 to 10.0)	4.10 (–1.73 to 9.94)
>55 – ≤60	49	92	1.08 (0.67–1.72)	0.99 (0.60–1.64)	3.31 (–2.83 to 9.46)	2.25 (–3.84 to 8.33)
>60 – ≤65	42	104	1.42 (0.88–2.29)	1.49 (0.89–2.49)	4.12 (–1.97 to 10.2)	5.05 (–1.02 to 11.1)
>65	31	83	1.53 (0.91–2.59)	1.44 (0.84–2.48)	9.14 (2.61 to 15.7)	8.03 (1.73 to 14.3)
BMI (kg/m2)						
<20	12	29	0.80 (0.45–1.86)	0.97 (0.46–2.04)	1.12 (–3.25 to 5.50)	1.47 (–2.73 to 5.68)
≥20– <25	92	244	1.00	1.00	Baseline	Baseline
≥25– <30	82	177	0.26 (0.57–1.16)	0.79 (0.55–1.14)	−1.55 (–3.74 to 0.63)	−1.73 (–3.84 to 0.39)
≥30	48	43	0.34 (0.21–0.54)	0.31 (0.19–0.52)	−6.84 (–9.97 to –3.72)	−6.98 (–10.0 to –3.94)
Oral contraception						
No	126	240	1.00	1.00	Baseline	Baseline
Yes	108	253	1.23 (0.90–1.68)	1.49 (1.04–2.13)	2.84 (–1.13 to 6.80)	5.40 (1.17 to 9.62)
Menopausal status						
Pre	67	120	1.00	1.00	Baseline	Baseline
Peri/post	167	373	1.25 (0.88–1.77)	1.22 (0.70–2.12)	4.42 (–0.12 to 8.95)	3.62 (–3.10 to 10.3)
MHT** (n = 537)						
No	135	287	1.00	1.00	Baseline	Baseline
Yes	30	85	1.33 (0.84–2.12)	1.37 (0.85–2.23)	−1.52 (–4.53 to 1.48)	−1.55 (–4.50 to 1.40)
Smoking status						
Never	97	209	1.00	1.00	Baseline	Baseline
Ex	66	135	0.95 (0.65–1.39)	1.06 (0.71–1.58)	−0.58 (–5.44 to 4.28)	0.85 (–3.91 to 5.62)
Current	71	149	0.97 (0.67–1.41)	1.03 (0.70–1.52)	1.81 (–2.93 to 6.54)	2.42 (–2.18 to 7.03)
Alcohol consumption						
Zero	25	32	1.00	1.00	Baseline	Baseline
<15 g/day	311	315	1.73 (0.99–3.03)	1.92 (1.07–3.45)	8.60 (1.10 to 16.1)	9.31 (2.05 to 16.6)
15 – 30 g/day	30	70	1.82 (0.93–3.58)	2.16 (1.06–4.41)	9.20 (0.34 to 18.1)	10.7 (2.03 to 19.3)
>30 g/day	8	12	1.17 (0.42–3.30)	1.44 (0.48–4.33)	2.38 (–11.5 to 16.3)	4.51 (–9.01 to 18.0)
Infrequent	29	64	1.72 (0.87–3.41)	2.01 (0.99–4.08)	9.41 (0.42 to 18.4)	10.9 (2.21 to 19.5)
Baseline season						
Spring	95	104	1.00	1.00	Baseline	Baseline
Summer	39	164	3.84 (2.46–6.00)	3.89 (2.48–6.12)	20.3 (15.2 to 25.5)	20.1 (14.9 to 25.2)
Autumn	39	110	2.58 (1.63–4.08)	2.52 (1.59–4.00)	11.0 (5.45 to 16.6)	10.5 (4.94 to 16.1)
Winter	61	115	1.72 (1.14–2.61)	1.71 (1.12–2.60)	7.04 (1.72 to 12.4)	6.80 (1.47 to 12.1)
Education						
O–level college	158	338	1.00	1.00	Baseline	Baseline
A–level college	23	34	0.69 (0.39–1.21)	0.70 (0.39–1.27)	−5.87 (–13.3 to 1.61)	−5.63 (–12.9 to 1.62)
University	53	121	1.07 (0.73–1.55)	1.17 (0.78–1.74)	−2.31 (–7.02 to 2.40)	−1.10 (–5.77 to 3.57)
Socio–economic index						
Manual	93	181	1.00	1.00	Baseline	Baseline
Non–manual	120	276	1.18 (0.85–1.64)	1.24 (0.88–1.75)	−0.37 (–4.57 to 3.83)	0.20 (–3.87 to 4.27)
Employer	20	36	0.93 (0.51–1.69)	0.88 (0.47–1.65)	−4.91 (–12.7 to 2.94)	−4.97 (–12.6 to 2.62)
Born in Sweden						
Yes	200	457	1.00	1.00	Baseline	Baseline
No	34	36	0.46 (0.28–0.76)	0.44 (0.26–0.74)	−10.6 (–17.3 to –3.96)	−10.5 (–16.9 to –4.08)

* Adjusted for age, season and time in freezer. ** Only in peri/postmenopausal.

The association between 25OHD3 and alcohol showed a bimodal pattern where 25OHD3 quartiles 1 and 4 had the highest percentage of high consumers (>30 g/day), Table 1. Correspondingly, women who reported no alcohol consumption or were high consumers had low 25OHD3 levels, Table 2, and this pattern was confirmed in the dichotomised analysis and in the continuous analysis, Table 3.

The highest concentration of 25OHD3 was seen during the summer and in the autumn, Table 1. This was confirmed in both the logistic regression analysis and in the linear regression analysis, Table 3. Women with relatively low vitamin D levels were more often born abroad, Table 1. This association was confirmed in the dichotomised analysis and in the continuous analysis, Table 3. Vitamin D was not associated with menopausal status, use of MHT, smoking status, education and socio-economic index in this study.

Most results were similar following inclusion of dietary intake of total vitamin D in the logistic regression analysis, but the OR s for oral contraception changed from 1.49 (1.04-2.13) to 1.40 (0.97-2.02), for Born in Sweden from 0.44 (0.26-0.74) to 0.51 (0.30-0.89 and for menopausal status from 1.22 (0.70-2.12) to 1.28 (0.72-2.25).

### Biochemical variables

Women with relatively high 25OHD3 levels had more often high albumin levels, Table 4, but the association did not reach statistical significance when 25OHD3 levels were compared in different albumin quartiles, Table 5, or in the dichotomised analysis, Table 6. However, there was a statistically significant positive association in the linear regression analysis, Table 6. 25OHD3 was also positively associated with creatinine, phosphate and calcium in all analyses, Tables 4, 5, and 6; although the association was borderline significant concerning phosphate in the adjusted dichotomised analysis, Table 6. In all analyses, there was an inverse association between 25OHD3 and PTH concentrations, Tables 4, 5, and 6. All crude and adjusted analyses were similar.

**Table 4:** Serum vitamin D quartiles in relation to biochemical parameters and dietary intake

	**Serum vitamin D quartile [nmol/L]**				
	**1 [18–69] n = 179**	**2 [70–86] n = 187**	**3 [87–104] n = 177**	**4 [105–173] n = 184**	**All n = 727**
Column percent*					
Albumin					
1 (30.0–39.0)	30.2	25.7	26.0	23.4	26.3
2 (39.1–40.9)	21.2	24.6	20.3	23.9	22.6
3 (41.0–42.2)	22.9	26.7	31.6	20.1	25.3
4 (42.3–50.3)	22.9	22.5	21.5	32.1	24.8
Creatinine					
1 (42–58)	30.2	27.8	26.0	20.1	26.0
2 (59–64)	22.3	24.6	24.3	21.2	23.1
3 (65–71)	28.5	24.6	23.2	24.5	25.2
4 (72–130)	16.2	22.5	26.0	33.7	24.6
Phosphate					
1 (0.66–1.07)	29.6	21.4	24.9	23.9	24.9
2 (1.08–1.19)	29.6	26.7	24.9	19.0	25.0
3 (1.20–1.32)	23.5	20.3	28.8	24.5	24.2
4 (1.33–2.00)	14.5	31.0	20.9	32.1	24.8
PTH					
1 (0.38–2.63)	22.3	17.6	26.0	33.7	24.9
2 (2.64–3.55)	18.4	27.3	29.9	22.3	24.5
3 (3.56–4.89)	23.5	28.3	21.5	25.0	24.6
4 (4.90–30.60)	34.1	25.1	22.6	18.5	25.0
Calcium					
1 (2.18–2.34)	35.8	26.2	22.6	24.5	27.2
2 (2.35–2.38)	19.6	24.6	23.2	17.4	21.2
3 (2.39–2.44)	26.8	23.5	27.7	26.1	26.0
4 (2.45–2.77)	15.1	24.6	26.0	32.1	24.5
Vitamin D– dietary					
1 (0.83–7.19)	30.2	16.6	15.8	17.4	19.9
2 (2.67–10.17)	21.8	21.4	16.9	20.1	20.1
3 (2.66–11.86)	15.6	25.1	21.5	17.4	19.9
4 (3.54–12.57)	19.0	17.1	21.5	22.8	20.1
5 (5.14–22.83)	13.4	19.8	24.3	22.3	19.9
Calcium– dietary					
1 (275.8–1203.8)	21.8	19.3	16.9	21.7	19.9
2 (435.5–1712.1)	19.0	23.0	19.2	19.0	20.1
3 (460.7–1669.4)	17.3	18.7	23.7	20.1	19.9
4 (636.6–2399.4)	21.2	*16.6*	22.6	20.1	20.1
5 (779.5–2791.5)	20.7	22.5	17.5	19.0	19.9
Vitamin D–total**					
1 (0.83–9.31)	31.8	16.0	16.9	15.2	19.9
2 (2.66–11.86)	20.7	25.7	12.4	21.2	20.1
3 (3.97–12.57)	18.4	18.2	23.2	20.1	19.9
4 (4.84–16.55)	12.8	20.9	24.3	22.3	20.1
5 (6.47–37.01)	16.2	19.3	23.2	21.2	19.9
Calcium–total**					
1 (275.8–1203.8)	22.9	18.7	15.8	22.3	19.9
2 (435.5–1712.1)	17.9	23.0	23.2	16.3	20.1
3 (532.6–2089.9)	18.4	19.8	20.9	20.7	19.9
4 (723.5–2399.4)	22.3	17.1	19.8	21.2	20.1
5 (782.7–2986.8)	18.4	21.4	20.3	19.6	19.9

* Due to missing values, percentages do not always add up to 100%. ** Intake from diet and supplements.

**Table 5:** Mean serum vitamin D levels in relation to biochemical parameters and dietary intake

**Factor**	**No.**	**Mean (95% CI)**	T–test (p–value*)	**ANOVA (p–value)**
Albumin				
1	191	87.0 (82.5–91.6)	Ref.	
2	164	91.2 (86.4–95.9)	0.344	
3	184	88.7 (84.1–93.3)	0.730	
4	180	91.7 (87.0–96.4)	0.058	0.076
Creatinine				
1	189	88.4 (84.0–92.9)	Ref.	
2	168	88.2 (83.5–92.8)	0.337	
3	183	87.1 (82.2–91.9)	0.581	
4	179	94.5 (89.8–99.2)	0.001	0.003
Phosphate				
1	181	89.8 (84.3–95.3)	Ref.	
2	182	83.5 (79.3–87.8)	0.177	
3	176	91.3 (86.7–95.8)	0.308	
4	180	93.5 (89.1–97.8)	0.012	<0.001
PTH				
1	181	95.3 (90.3–100.3)	Ref.	
2	178	89.7 (85.5–93.8)	0.035	
3	179	89.0 (84.4–93.6)	0.029	
4	182	84.5 (79.7–89.3)	<0.001	0.002
Calcium				
1	198	86.6 (82.1–91.1)	Ref.	
2	154	85.7 (81.0–90.3)	0.425	
3	189	90.9 (86.1–95.8)	0.067	
4	178	93.8 (89.2–98.3)	<0.001	0.001
Vitamin D–dietary				
1	145	81.1 (76.5 – 85.6)	Ref.	
2	146	86.7 (82.3 – 91.1)	0.08	
3	145	88.3 (84.1 – 92.6)	0.02	
4	146	92.1 (87.4 – 96.7)	0.001	
5	145	93.1 (88.9 – 97.4)	<0.001	0.001
Calcium–dietary				
1	145	87.3 (82.7 – 91.9)	Ref.	
2	146	88.2 (83.8 – 92.6)	0.79	
3	145	90.1 (85.7 – 94.6)	0.39	
4	146	88.0 (83.4 – 92.7)	0.83	
5	145	87.6 (83.4 – 91.9)	0.92	0.92
Vitamin D–total**				
1	145	79.9 (75.3 – 84.6)	Ref.	
2	146	86.0 (81.8 – 90.2)	0.06	
3	145	90.9 (86.2 – 95.7)	0.001	
4	146	93.5 (89.2 – 97.8)	<0.001	
5	145	90.9 (86.7 – 95.0)	0.001	<0.001
Calcium–total**				
1	145	87.6 (82.8 – 92.3)	Ref.	
2	146	87.4 (83.2 – 91.7)	0.97	
3	145	89.4 (84.8 – 94.0)	0.59	
4	146	88.2 (83.6 – 92.8)	0.85	
5	145	88.8 (84.7 – 92.9)	0.70	0.97

* Compared to reference (p-value). ** Intake from diet and supplements.

**Table 6:** **Crude and adjusted odds ratios (OR) for high (> 75 nmol/L) *vs*. low (≤75 nmol/L) vitamin D levels, and regression coefficients (βi) from multiple regression analysis, in relation to biochemical parameters and dietary intake**

**Factor**	**Low (n)**	**High (n)**	**Crude OR (95% CI)**	Adjusted*OR (95% CI)	**βi (95% CI)**	Adjusted* βi \(95% CI)
Albumin						
1	68	123	1.00	1.00	Baseline	Baseline
2	50	114	1.26 (0.81–1.97)	1.15 (0.72– 1.83)	2.66 (–3.02 to 8.34)	1.55 (–3.92 to 7.02)
3	55	129	1.30 (0.84–2.00)	1.22 (0.78– 1.92)	0.94 (–4.57 to 6.45)	0.18 (–5.13 to 5.48)
4	56	124	1.22 (0.79–1.89)	1.27 (0.81– 2.00)	5.59 (0.05 to 11.13)	6.73 (1.39 to 12.1)
Creatinine						
1	66	123	1.00	1.00	Baseline	Baseline
2	55	113	1.10 (0.71– 1.71)	1.07 (0.68– 1.68)	2.40 (–3.23 to 8.02)	2.47 (–2.93 to 7.86)
3	69	114	0.89 (0.58–1.35)	0.84 (0.54– 1.30)	1.61 (–3.89 to 7.11)	1.38 (–3.90 to 6.66)
4	39	140	1.93 (1.21– 3.06)	1.70 (1.05– 2.76)	9.11 (3.58 to 14.6)	6.97 (1.57 to 12.4)
Phosphate						
1	62	119	1.00	1.00	Baseline	Baseline
2	69	113	0.85 (0.56–1.31)	0.75 (0.48– 1.18)	−3.79 (–9.34 to 1.76)	−4.71 (–10.0 to 0.58)
3	54	122	1.18 (0.76–1.83)	1.12 (0.71– 1.78)	3.04 (–2.56 to 8.64)	2.81 (–2.54 to 8.16)
4	44	136	1.61 (1.02–2.55)	1.54 (0.96– 2.48)	7.33 (1.77 to 12.9)	6.93 (1.63 to 12.2)
PTH						
1	51	130	1.00	1.00	Baseline	Baseline
2	45	133	1.16 (0.73–1.85)	1.26 (0.77– 2.05)	−5.83 (–11.4 to –0.23)	−4.76 (–10.1 to 0.62)
3	56	123	0.86 (0.55–1.36)	0.83 (0.52– 1.32)	−6.28 (–11.9 to –0.70)	−6.71 (–12.1 to –1.34)
4	77	105	0.54 (0.35–0.83)	0.59 (0.37– 0.93)	−11.8 (–17.3 to –6.22)	−9.96 (–15.3 to –4.61)
Calcium						
1	80	118	1.00	1.00	Baseline	Baseline
2	47	107	1.55 (0.99–2.41)	1.66 (1.04 –2.64)	2.19 (–3.50 to 7.88)	2.80 (–2.63 to 8.24)
3	57	132	1.57 (1.03–2.39)	1.64 (1.05– 2.54)	5.10 (–0.29 to 10.5)	5.79 (0.63 to 11.0)
4	45	133	2.00 (1.29–3.12)	2.18 (1.37– 3.47)	10.2 (4.78 to 15.7)	11.1 (5.79 to 16.3)
Vitamin D–dietary						
1	68	77	1.00	1.00	Baseline	Baseline
2	51	95	1.65 (1.03– 2.64)	1.70 (1.04– 2.78)	5.64 (–0.57 to 11.9)	5.35 (–0.64 to 11.3)
3	36	109	2.67 (1.63– 4.40)	2.92 (1.74– 4.91)	7.29 (1.07 to 13.5)	7.50 (1.50 to 13.5)
4	41	105	2.26 (1.39–3.68)	2.52 (1.51– 4.19)	11.0 (4.82 to 17.2)	11.5 (5.47 to 17.5)
5	38	107	2.28 (1.52–.07)	2.68 (1.58– 4.57)	12.1 (5.86 to 18.3)	11.8 (5.58 to 17.9)
Calcium–dietary						
1	53	92	1.00	1.00	Baseline	Baseline
2	48	98	1.18 (0.73–1.91)	1.20 (0.73– 1.99)	0.87 (–5.41 to 7.16)	0.90 (–5.16 to 6.95)
3	39	106	1.57 (0.95–2.58)	1.63 (0.97– 2.73)	2.79 (–3.51 to 9.08)	3.08 (–2.98 to 9.13)
4	46	100	1.25 (0.77–2.04)	1.36 (0.82– 2.27)	0.71 (–5.58 to 7.00)	1.26 (–4.81 to 7.34)
5	48	97	1.16 (0.72–1.89)	1.23 (0.74– 2.03)	0.30 (–6.00 to 6.60)	0.67 (–5.40 to 6.74)
Vitamin D–total**						
1	70	75	1.00	1.00	Baseline	Baseline
2	46	100	2.03 (1.26–3.27)	2.05 (1.25– 3.38)	6.08 (–0.12 to 12.3)	5.63 (–0.31 to 11.6)
3	39	106	2.54 (1.55–4.14)	3.01 (1.79– 5.05)	11.0 (4.80 to 17.2)	12.1 (6.09 to 18.0)
4	40	106	2.47 (1.52–4.03)	2.96 (1.76– 4.99)	13.5 (7.36 to 19.7)	14.8 (8.81 to 20.8)
5	39	106	2.54 (1.55– 4.14)	2.84 (1.69– 4.78)	10.9 (4.72 to 17.1)	11.5 (5.50 to 17.5)
Calcium–total**						
1	55	90	1.00	1.00	Baseline	Baseline
2	46	100	1.33 (0.82–2.16)	1.36 (0.82– 2.26)	−0.13 (–6.42 to 6.15)	−0.15 (–6.19 to 5.90)
3	41	104	1.55 (0.95–2.54)	1.63 (0.97– 2.71)	1.82 (–4.48 to 8.12)	2.46 (–3.61 to 8.53)
4	49	97	1.21 (0.75–1.96)	1.34 (0.81– 2.22)	0.63 (–5.65 to 6.92)	1.68 (–4.39 to 7.75)
5	43	102	1.45 (0.89–2.37)	1.52 (0.91– 2.52)	1.22 (–5.07 to 7.52)	1.43 (–4.63 to 7.50)

* Adjusted for age, season and time in freezer for all factors in the table. ** Intake from diet and supplements.

### Dietary intake

High serum concentrations of 25OHD3 were associated with a high intake of vitamin D, Table 4. This finding was confirmed comparing 25OHD3 levels in different categories of vitamin D intake, Table 5, and in both the dichotomised and continuous analysis, Table 6. The use of dietary intake of vitamin D from the diet alone, or from diet and supplements, showed similar results. Contrary to this, there was no statistically significant association between intake of Calcium and 25OHD3 levels, Tables 4, 5, and 6.

## Discussion

This cross-sectional study including 727 females found that serum levels of 25OHD3 are positively associated with age, oral contraceptive use, moderate alcohol consumption, and blood collection during summer / autumn, a high dietary intake of vitamin D and serum concentrations of creatinine, phosphate and calcium. Serum levels of 25OHD3 were inversely associated with obesity, being born outside Sweden and with serum levels of PTH.

A positive association between serum levels of 25OHD3 and advanced age has previously also been reported by Moan et al. [32]. However, others have found vitamin D deficiency to be more prevalent among elderly people [33], which could be caused by poor production in the skin [34], decreased intestinal absorption [35], and/or impaired renal function [36]. A reason for the difference between our and most previous studies may be that the current study only included females up until the age of 72 years, and deficiency related to high age may not be apparent until later. Another possible reason to the results seen in the present study may be that older women in this cohort consume relatively more vitamin D. We found no association between menopausal status and serum levels of 25OHD3, although postmenopausal women had slightly higher levels.

A positive association between the use of OC and serum level of 25OHD3 was found in the present study and there was also a tendency for a positive association between MHT and high serum levels of 25OHD3. This is in line with several previous studies on 25OHD3 in relation to OC and other exogenous oestrogens [37-41]. A potential explanation may be that oestrogen increases the levels of 25OHD3-binding proteins [42].

We found no strong association between socioeconomic status and serum levels of 25OHD3, although serum levels of 25OHD3 were slightly higher in women with a non-manual vs. a manual employment.

The present study shows a positive association between high serum levels of 25OHD3 and moderate alcohol consumption, as opposed to high alcohol consumption and zero consumption. This observation is in accordance with a recent study finding that moderate alcohol consumers have a relatively high intake of vitamin D as compared to zero-consumers [43]. Differences with regard to dietary intake may be an explanation behind the low vitamin D levels in zero-consumers, for example vegetarians may have a relatively low intake of vitamin D [44] and it is possible to hypothesize that they consume less alcohol as compared to other groups. In general, the concentration of vitamin D has shown to be reduced among high consumers of alcohol. This could be due to a variation in the dietary intake of vitamin D during different drinking periods with marked reduction during hard-drinking weeks [45].

Another factor closely related to alcohol consumption is smoking, but in the present study there was no association between smoking habits and serum levels of 25OHD3, and smoking is not likely to explain the association seen in the present analysis.

We found that obesity was associated with relatively low serum levels of 25OHD3 and this is similar with several other studies [46-49]. A potential explanation is that poor dietary habits are related to increased BMI [50], and this may also lead to a lower intake of vitamin D in obese women as shown by Tidwell et al. [51]. Adipose tissue contains vitamin D receptors which hypothetically could lead to a sequestration of serum levels of 25OHD3 in obese individuals [52]. Moreover, obesity is more prevalent among people with lower education level [53], and this may be an indirect explanation for the results in this study as women with a manual vs. non-manual work had slightly lower serum levels of 25OHD3. However, educational levels per se as measured in the current study were not associated with serum levels of 25OHD3.

Women born abroad had lower serum levels of 25OHD3 in our report. Different geographical locations [54] and ethnic backgrounds [55,56] have, indeed, been correlated to the vitamin D level. Possible explanations could be related to cultural tradition with traditional dress style other than western dress [57], colour of the skin because darker skin has less capacity to make vitamin D from sunlight [58,59], or a genetic impact on serum vitamin D status probably through the skin synthesis of vitamin D [60]. Another explaining factor is variation in vitamin D intake between different ethnic groups as vitamin D intake is comparatively high in Scandinavian countries [61]. In this group ultraviolet light may not be the only key in the prevention of different condition but the role of dietary vitamin D intake, supplement vitamin D and calcium come into play [62-64]. In our study, the highest levels of serum 25OHD3 were seen during the summer and in the autumn and these are in agreement with previous studies [65-69]. This is also to be expected as during this season the solar UV radiation reaches adequate levels to have a sufficient vitamin D production (ibid).

We found a positive association between serum levels of 25OHD3, creatinine, phosphate and calcium, and an inverse association with PTH. This has also been seen in other studies [70], and follows the à priori hypothesis given the physiology for vitamin D metabolism. A sufficient concentration of vitamin D [71], is essential for bone mineralization and bone turnover [72] and to maintain an optimal vitamin D level requires a close physiological cooperation between vitamin D, calcium and PTH levels [73,74].

The positive association between intake of vitamin D and levels of 25OHD3 in this study was expected and has also been reported by others [75,76]. There was no strong association between calcium intake and levels of 25OHD3 and this has been reported by others [77].

Our study has several methodological strengths. Serum samples were collected in a standardized way and stored at −80°C, laboratory methods had low CVs, and there were a relatively large number of subjects, i.e., good statistical power. The inclusion of both life-style factors and biochemical parameters are additional strengths. However, some methodological questions need to be discussed. It may be questioned whether it is appropriate to use a single determination for levels of vitamin D. However, it is reassuring that a recent cohort study that measured 25OHD3 at 2 times, 3 years apart, found a high correlation between levels. Information on life-style and diet was collected using various questionnaires and this may lead to misclassification. However, validity and reproducibility is high for both the socioeconomic questionnaire and the dietary assessment method [23,25].

The results were adjusted for age, season and storage time, and these factors ought not to have confounded the results. It was considered appropriate to adjust only for these factors, as we wanted to identify groups with low vs. high vitamin D levels, rather than to determine what factors that affected vitamin D levels the most. Concerning dietary factors, the residual method was used, which takes into account differences with regard to total energy intake, i.e. the amount of the total dietary intake.

A valid question is whether the associations observed in this cohort can be representative of the whole population. Although in this study only 40% of the populations have participated and as we have no information about exposure to the studied risk factors in women outside this cohort, observed means and the prevalence for studied factors may not be applicable to all age groups or to the general population. However, as there was a wide distribution of 25OHD3 and other determinants, it was possible to make internal comparisons between subjects. We consider that our estimations of odds ratios were less sensitive to a potential selection bias.

## Conclusions

The present population-based cohort study including 727 females found a positive association between serum levels of 25OHD3 and age, oral contraceptive use, moderate alcohol consumption, blood collection during summer/ autumn, creatinine, phosphate, calcium, and a high intake of vitamin D. Low vitamin D levels were associated with obesity, being born outside Sweden and high PTH levels. The study may have implications e. g. for dietary recommendations. However, the analysis is a cross-sectional study and it is difficult to suggest lifestyle recommendations as cause- effect relationships are not clear.

## Competing interests

The authors declare that they have no competing interests.

## Authors’ contributions

Planning and design: MA, JMal, JMan, EW, LZ. Supervision of laboratory analyses: JMal. Statistical analysis: JMan, LZ. Interpretation of results: MA, JMal, JMan, EW, LZ. Draft of paper: LZ. Revision of paper: MA, JMal, JMan, EW, LZ. Final approval of paper: MA, JMal, JMan, EW, LZ. All authors read and approved final manuscript.

## Pre-publication history

The pre-publication history for this paper can be accessed here:

http://www.biomedcentral.com/1472-6874/13/33/prepub
